# Yeast-based assays for screening 11β-HSD1 inhibitors

**DOI:** 10.1186/s12934-016-0450-6

**Published:** 2016-03-15

**Authors:** Rosario Vanella, Roberta Callari, Anna Weston, Harald Heider, Markus S. Schwab, Eric Kübler

**Affiliations:** University of Applied Sciences Northwestern Switzerland, Gründenstrasse 40, 4132 Muttenz, Switzerland; Evolva SA, Duggingerstrasse 23, 4153 Reinach, Switzerland

**Keywords:** 11β-HSD1 inhibition, Metabolic syndrome, Skin ageing, Yeast based assay, TEV protease, TIPI mechanism, Pdr5p ABC transporter

## Abstract

**Background:**

Intracellular metabolism of glucocorticoid hormones plays an important role in the pathogenesis of metabolic syndrome and regulates, among many physiological processes, collagen metabolism in skin. At the peripheral level the concentration of active glucocorticoids is mainly regulated by the 11β-hydroxysteroid dehydrogenase 1 (11β-HSD1) enzyme, involved in the conversion of cortisone into the biologically active hormone cortisol. Cortisol interacts with the glucocorticoid receptor and regulates the expression of different classes of genes within the nucleus. Due to its implication in glucocorticoid metabolism, the inhibition of 11β-HSD1 activity has become a dominant strategy for the treatment of metabolic syndrome. Moreover, inhibitors of this target enzyme can be used for development of formulations to counteract skin ageing. Here we present the construction of two yeast cell based assays that can be used for the screening of novel 11β-HSD1 inhibitors.

**Results:**

The yeast *Saccharomyces cerevisiae* is used as a host organism for the expression of human 11β-HSD1 as well as a genetically encoded assay system that allows intracellular screening of molecules with 11β-HSD1 inhibitory activity. As proof of concept the correlation between 11β-HSD1 inhibition and fluorescent output signals was successfully tested with increasing concentrations of carbenoxolone and tanshinone IIA, two known 11β-HSD1 inhibitors. The first assay detects a decrease in fluorescence upon 11β-HSD1 inhibition, whereas the second assay relies on stabilization of yEGFP upon inhibition of 11β-HSD1, resulting in a positive read-out and thus minimizing the rate of false positives sometimes associated with read-outs based on loss of signals. Specific inhibition of the ABC transporter Pdr5p improves the sensitivity of the assay strains to cortisone concentrations by up to 60 times.

**Conclusions:**

Our yeast assay strains provide a cost-efficient and easy to handle alternative to other currently available assays for the screening of 11β-HSD1 inhibitors. These assays are designed for an initial fast screening of large numbers of compounds and enable the selection of cell permeable molecules with target inhibitory activity, before proceeding to more advanced selection processes. Moreover, they can be employed in yeast synthetic biology platforms to reconstitute heterologous biosynthetic pathways of drug-relevant scaffolds for simultaneous synthesis and screening of 11β-HSD1 inhibitors at intracellular level.

## Background

Glucocorticoids, such as cortisone and cortisol in human, are stress hormones with a vital role in regulation of metabolic and defence responses. They exert a role in many mammalian biological processes including regulation of energy metabolism, immune and inflammatory responses, cardiovascular homeostasis and the body’s responses to stress [1–3]. Several studies have also underlined the role of glucocorticoids in regulation of collagen homeostasis and wound healing processes [4, 5]. Research performed over the past 20 years has shed light on the involvement of glucocorticoid metabolism in the pathogenesis of obesity, a major risk factor for the metabolic syndrome, a collection of disorders including insulin resistance, dyslipidemia and hypertension [6].

A key step in the molecular elucidation of these biological processes and conditions has been the demonstration that glucocorticoid receptor activation in target tissues is not determined exclusively by circulating glucocorticoids but also by intra-cellular interconversion of active and inactive forms of the steroids [7]. Identification of this mechanism for local regulation of glucocorticoid action has prompted the concept that tissue-specific glucocorticoid excess, in the face of normal circulating concentrations, may contribute to metabolic syndromes [8] and plays an important role in the skin ageing process, reducing collagen metabolism and promoting skin atrophy [5].

The production of active glucocorticoid hormones at the intracellular level is regulated by the two hydroxysteroid dehydrogenase enzyme isoforms 11β-hydroxysteroid dehydrogenase 1 (11β-HSD1) and 11β-HSD2, microsomal enzymes belonging to the family of short chain dehydrogenases/reductases. The 11β-hydroxysteroid dehydrogenases (11β-HSDs) catalyse the interconversion of inactive cortisone to active cortisol and vice versa [9]. 11β-HSD2 is predominantly expressed in mineralocorticoid target tissues, such as salivary glands, colon and kidneys. Its role is to protect the non-selective mineralocorticoid receptor from the unspecific binding from cortisol, catalysing its conversion to cortisone [10].

11β-HSD1 acts in vivo mainly as a reductase, converting cortisone into the biologically active steroid cortisol in a NADPH dependent manner [11]. The intracellular co-localization of 11β-HSD1 with hexose-6-phosphate dehydrogenase has a fundamental role in providing NADPH as cofactor driving the reductase activity of 11β-HSD1, rather than the dehydrogenase activity [12]. In vitro 11β-HSD1 has a *Km* in the µM range for both cortisone and cortisol [13]. The enzyme is mostly expressed in liver, fat, gonadal tissue and the central nervous system, where it amplifies local glucocorticoid concentrations [9]. Inside the cells cortisol interacts with the cytosolic glucocorticoid receptor (GR), prompting receptor dimerization and translocation to the nucleus. Receptor dimers then bind to the glucocorticoid response element (GRE) in target genes, leading to regulation of their transcription [14]. The main actions of GR-mediated glucocorticoid stimulation include anti-inflammatory and immunosuppressive effects, the regulation of the metabolism of glucose, and the differentiation of adipose tissue [15].

Genetic models developed in mice suggested that overexpression of 11β-HSD1 is a factor involved in the development of central obesity and type 2 diabetes [16]. Moreover 11β-HSD1 knock-out mice showed enhanced glucose tolerance and improved lipid and lipoprotein profiles [17]. Increased cortisol levels due to overexpression of 11β-HSD1 in aged skin were demonstrated to be common features of mammalian ageing processes. Conversely 11β-HSD1 knock-out mice showed improved age-induced dermal atrophy and structural disorganization with increased collagen density and faster wound healing processes [18].

In the light of this evidence, specific inhibition of 11β-HSD1 has been proposed as a crucial strategy for treatment of metabolic syndrome and for the stimulation of collagen organization and wound healing in elderly and diabetic individuals [15, 18, 19].

In recent years many research groups have developed different strategies for the discovery of new candidate molecules exhibiting selective inhibitory effects towards human 11β-HSD1. Most approaches for selection of candidate inhibitors use liver microsomes or recombinant mammalian cell lines expressing 11β-HSD1. Analyses of cortisone and cortisol contents are performed using Liquid Chromatography-Mass Spectrometry [20]. Other strategies involve the stable heterologous expression of 11β-HSD1 combined with β-galactosidase reporter constructs under control of glucocorticoid response elements [21]. Even though these strategies represent powerful tools to filter and select active compounds from large libraries of molecules, they often require the use of hazardous radioactive substances or the use of colorimetric materials that can interfere with the tested molecules. Moreover, they require expensive instrumentation and expertise.

The baker’s yeast *Saccharomyces cerevisiae* has emerged in the last decades as a powerful organism for the study of many human target enzymes. The deep genetic information available on this system has allowed it to become an increasingly popular model for pharmacological and/or drug discovery studies that can be used to analyse the effects of drugs in vivo during initial stages, before tests are performed in mammalian systems [22, 23]. Compared to higher eukaryotes, yeast cells are more economical, easier to grow and can be genetically manipulated with greater ease. Furthermore, yeast cells are generally freely accessible to small molecules and can even be engineered to increase permeability if necessary. Hence, the availability of well-established genomic methods and resources makes budding yeast an extremely valuable asset for the screening of molecules with possible pharmaceutical applications [24].

In order to develop an efficient and reliable alternative for screening large numbers of candidate inhibitors of 11β-HSD1, two yeast strains were constructed, functionally co-expressing the murine GR and the human 11β-HSD1 enzyme. In these strains, cortisone is taken up from the medium and converted—through the action of 11β-HSD1—to cortisol that then binds to the GR. In each of the two strains, the cortisol/GR activates a specific gene whose expression leads to the modulation of a yEGFP fluorescent signal. In one strain, the signal is converted into a positive output (stable fluorescence) in the presence of an 11β-HSD1 inhibitor; in the other strain inhibition of 11β-HSD1 leads to a loss of signal. To increase sensitivity of the assay systems the yeast multi drug efflux system and in particular the function of the yeast Pdr5 ATP-binding cassette (ABC) transporter in cortisone efflux was investigated and modified [25].

## Results

### Construction and validation of the assay strain RVY97

**Fig. 1:**
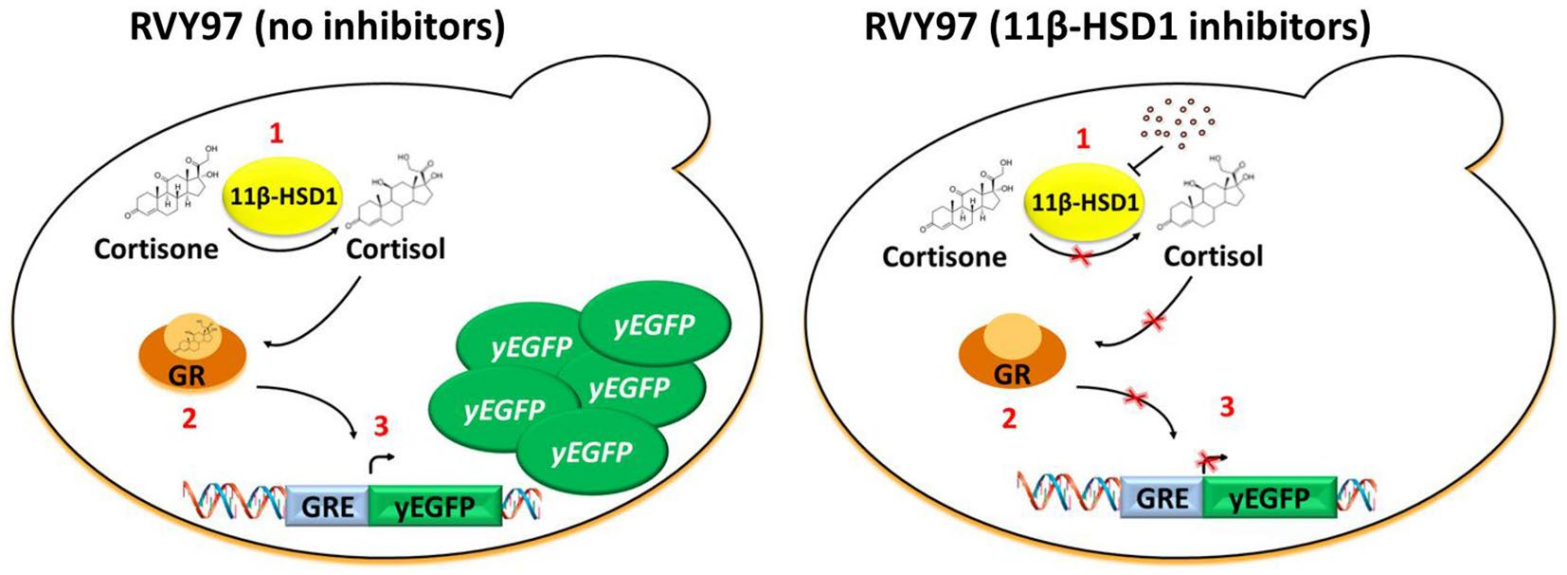
Schematic representation of the screening strain RVY97. In strain RVY97 the cortisone provided in the growth medium is converted to cortisol inside the cell by the enzyme 11β-HSD1. Cortisol binds the GR inducing assembly of an active complex that translocates to the nucleus. Through binding of the complex to the GRE, expression of the yEGFP reporter protein is triggered. In the presence of 11β-HSD1 inhibitors, cortisol is not produced, the GR is inactive and yEGFP not expressed

In order to develop a valid alternative to the biochemical and mammalian cell-based assays employed for the screening of 11β-HSD1 inhibitors we constructed the yeast strain RVY97, in which the activity of the 11β-HSD1 enzyme can be easily assayed via fluorescent output. The human 11β-HSD1 enzyme, heterologously expressed in yeast cells, reduces the cortisone provided in the growth medium, thereby converting it to cortisol, the active form of the steroid hormone. Cortisol binds to the glucocorticoid receptor, also heterologously expressed in yeast. The active complex translocates into the nucleus, promoting the expression of the yeast enhanced green fluorescent protein (yEGFP) via specific interaction with the glucocorticoid response element (GRE) upstream of the reporter gene. In this strain inhibition of 11β-HSD1 causes a decrease of the green fluorescence (Fig. 1).

**Fig. 2:**
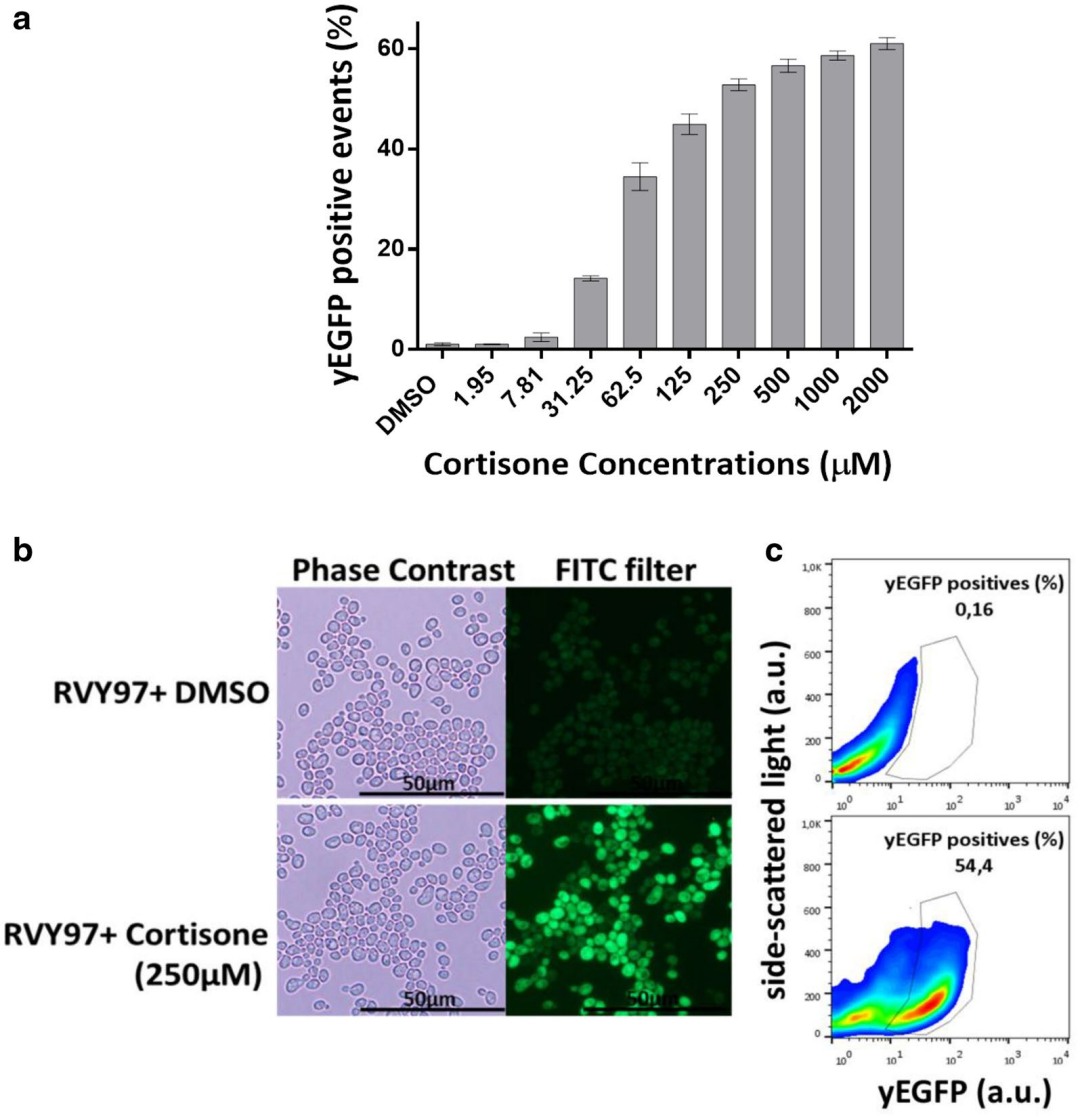
Effect of cortisone on yEGFP expression in the strain RVY97. **a** Increased cortisone concentrations led to an increase of yEGFP expression in a concentration dependent manner. The treated RVY97 population (10,000 events) was analysed by flow cytometry and the results are expressed in percentage of RVY97 cells showing yEGFP fluorescence. Data shown represent means ± standard deviations of three independent experiments. **b** Fluorescence microscopy of strain RVY97 showed that cortisone treated cells displayed a strong fluorescent response to the hormone. **c** Flow cytometry analysis of the strain RVY97 cultivated with DMSO (*upper panel*) and 250 μM cortisone (*lower panel*) enabled quantitative analysis of the hormone response of this specific strain. The gate highlights the RVY97 population positive for the yEGFP expression. All analyses were carried out 6 h after the addition of DMSO and cortisone, respectively. (a.u.): arbitrary unit

First, using flow cytometric analyses, the expression of yEGFP was assayed in strain RVY97 as a response to treatment with different cortisone concentrations, ranging from 1.95 to 2000 μM (Fig. 2a). As expected, green fluorescence in the treated population increased, due to the action of 11β-HSD1, with increasing concentrations of cortisone, whereas in the absence of hormone no fluorescent signal was detected. Concentrations of cortisone as low as 31.5 μM were effective, leading to the appearance of an unambiguous fluorescent signal connected to the activity of 11β-HSD1. However, higher concentrations of cortisone were more desirable due to the stronger induction of reporter protein expression. Expression of yEGFP, driven by 11β-HSD1-dependent conversion of cortisone to cortisol, rose significantly upon treatment with a range of cortisone concentrations between 60 and 500 μM. At higher concentrations a plateau of fluorescence intensity was reached (Fig. 2a). Of all hormone concentrations tested, 250 μM cortisone guaranteed the best compromise between intensity of the generated fluorescent signal and solubility of cortisone added to the yeast population. Flow cytometric analysis and fluorescence microscope images of RVY97 cells treated with DMSO (negative control) and 250 μM cortisone are shown in Fig. 2b, c.

To address the validity of the assay for screening of putative 11β-HSD1 inhibitors, a series of experiments with a synthetic inhibitor of this enzyme were performed. RVY97 strain was incubated with cortisone in presence of carbenoxolone (CBX), a synthetic hemisuccinyl ester derivative of glycyrrhetinic acid. Carbenoxolone has been shown to exert potent but non-selective 11β-HSD1 inhibitory activity and is frequently used as control in inhibition studies involving this target enzyme [26]. Carbenoxolone behaves as a competitor of cortisone, binding to the active domain of 11β-HSD1 [27, 28].

**Fig. 3:**
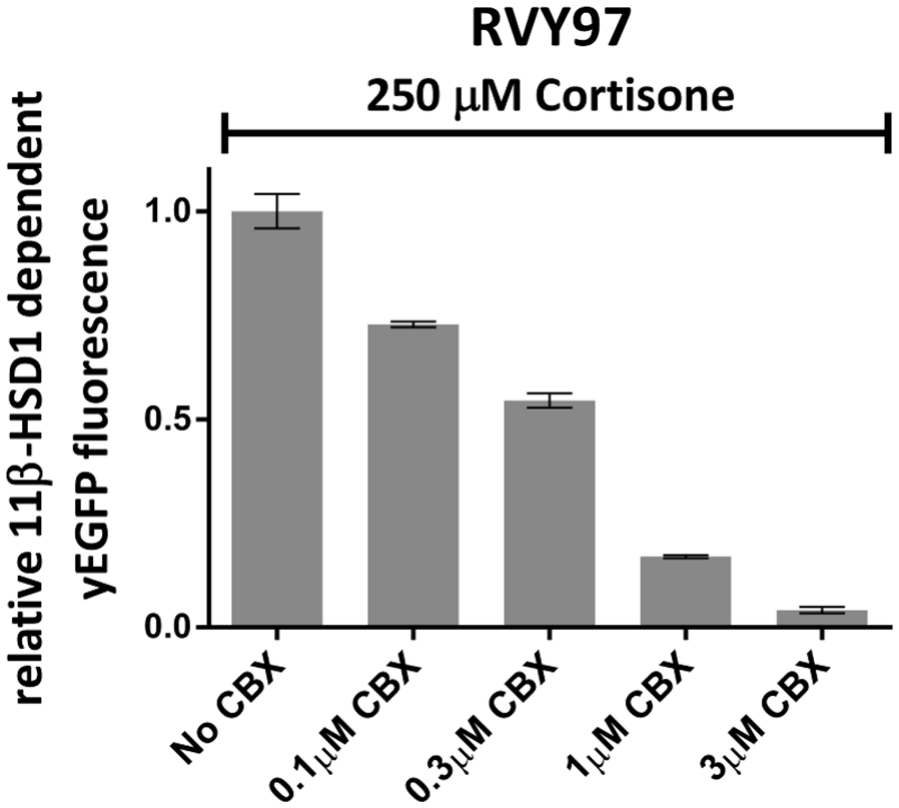
Effect of carbenoxolone (CBX) on 11β-HSD1 dependent response in the strains RVY97. 11β-HSD1 dependent yEGFP fluorescence of strain RVY97 treated with 250 μM cortisone and increasing CBX concentrations. Increasing concentration of carbenoxolone (up to 3 μM) led to a decreasing green fluorescence in the yeast population indicating a progressive inhibition of 11β-HSD1. The fluorescence of untreated cells (no CBX) was set at 1. Data shown represent means ± standard deviations of three independent experiments

In order to validate our assay with this molecule, different inhibitor concentrations ranging from 0.1 to 3 μM were added to 1 million RVY97 cells together with 250 μM of cortisone. Increasing concentrations of carbenoxolone caused a gradual loss of yEGFP expression, indicating a decreased activity of 11β-HSD1 in the population with a complete absence of fluoresce at a concentration of 3 μM of inhibitor, most probably associated with the complete inhibition of the enzyme (Fig. 3).

### Construction and validation of the assay strain RVY102

**Fig. 4:**
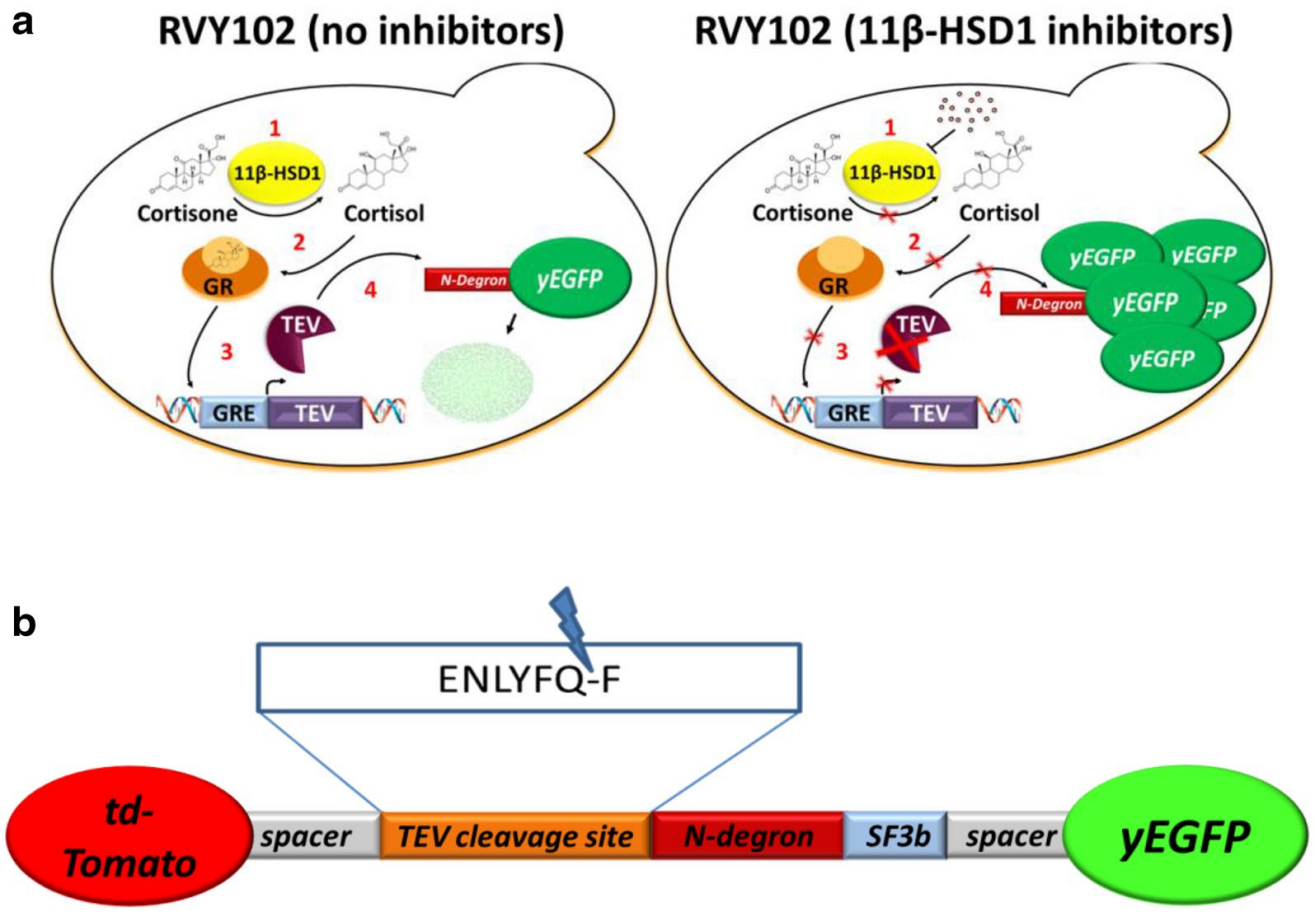
Schematic representation of the screening system RVY102. **a** Cortisone added to the growth medium enters RVY102 cells and is reduced to cortisol by the enzyme 11β-HSD1. The active complex formed by binding of cortisol to GR triggers expression of the TEV protease which binds to its cleavage site and cuts the reporter fluorescent protein that then undergoes degradation. Presence of an 11β-HSD1 inhibitor will stabilize the reporter protein. **b** *Graphic* representation of the reporter fusion protein expressed in strain RVY102. The fusion protein consists of the red tandem dimer (td) Tomato, the TEV recognition site, an N-degron destabilizing sequence, the SF3b155^381–424^ protein domain and the yeast enhanced green fluorescent protein (yEGFP). Once expressed, the TEV protease cuts at its cleavage site on the reporter protein, leading to exposure of the N-degron sequence and degradation of yEGFP. The TEV recognition sequence and the destabilizing amino acid (phenylalanine) are shown

A robust cell-based screening assay relies on strong correlation between an intracellular event and a univocal and easily detectable output. Screening systems with a positive readout guarantee the selection of hits from the analysis of large number of samples with minimum false positive results caused by unrelated intracellular events, such as impaired cellular viability or loss of an element of the assay system. To set up a more reliable assay for the screening of 11β-HSD1 inhibitors, a strain was constructed in which inhibition of 11β-HSD1 is connected to a positive fluorescent readout. In contrast to strain RVY97 where the activity of the enzyme is directly linked to the expression of a fluorescent reporter protein, in strain RVY102 the reduction of cortisone, driven by 11β-HSD1, is combined with a tobacco etch virus (TEV) protease induced protein instability (TIPI) mechanism [29] (Fig. 4a). This mechanism reversely combines the activity of the target enzyme to the stability of a constitutively expressed reporter protein.

The TIPI strategy exploits the presence of a TEV cleavage site in a reporter protein in order to induce its degradation. The reporter construct expresses a fluorescent fusion protein consisting of the red tandem dimer (td) Tomato, the TEV recognition site, an N-Degron destabilizing sequence, the SF3b155^381–424^ protein domain and the yEGFP [29] (Fig. 4b).

As shown in Fig. 4a, the expression of the TEV protease under control of the glucocorticoid response element is driven by the active complex formed after specific binding of cortisol to the glucocorticoid receptor. Expression of TEV leads to cleavage of the reporter protein and to the exposure of a dormant N-degron sequence. This destabilizing stretch of amino acids causes the C-terminal part of the reporter construct, including yEGFP, to undergo ubiquitination and proteasomal degradation [30]. The SF3b155^381–424^ protein domain binds strongly to the protein p14, fused to TEV, thus enhancing processivity of the protease [29]. The tdTomato red fluorescent protein is used for normalization purposes, since it does not undergo degradation upon TEV protease activity. On the other hand, the yEGFP reporter protein reverse monitors the TEV protease activity, as it is degraded upon TEV expression.

The described cascade in strain RVY102 directly couples the activity of 11β-HSD1 to yEGFP degradation. Inactivation of 11β-HSD1 caused by inhibitors, leads to stability of the reporter protein and detection of an intact green fluorescent signal in the population.

**Fig. 5:**
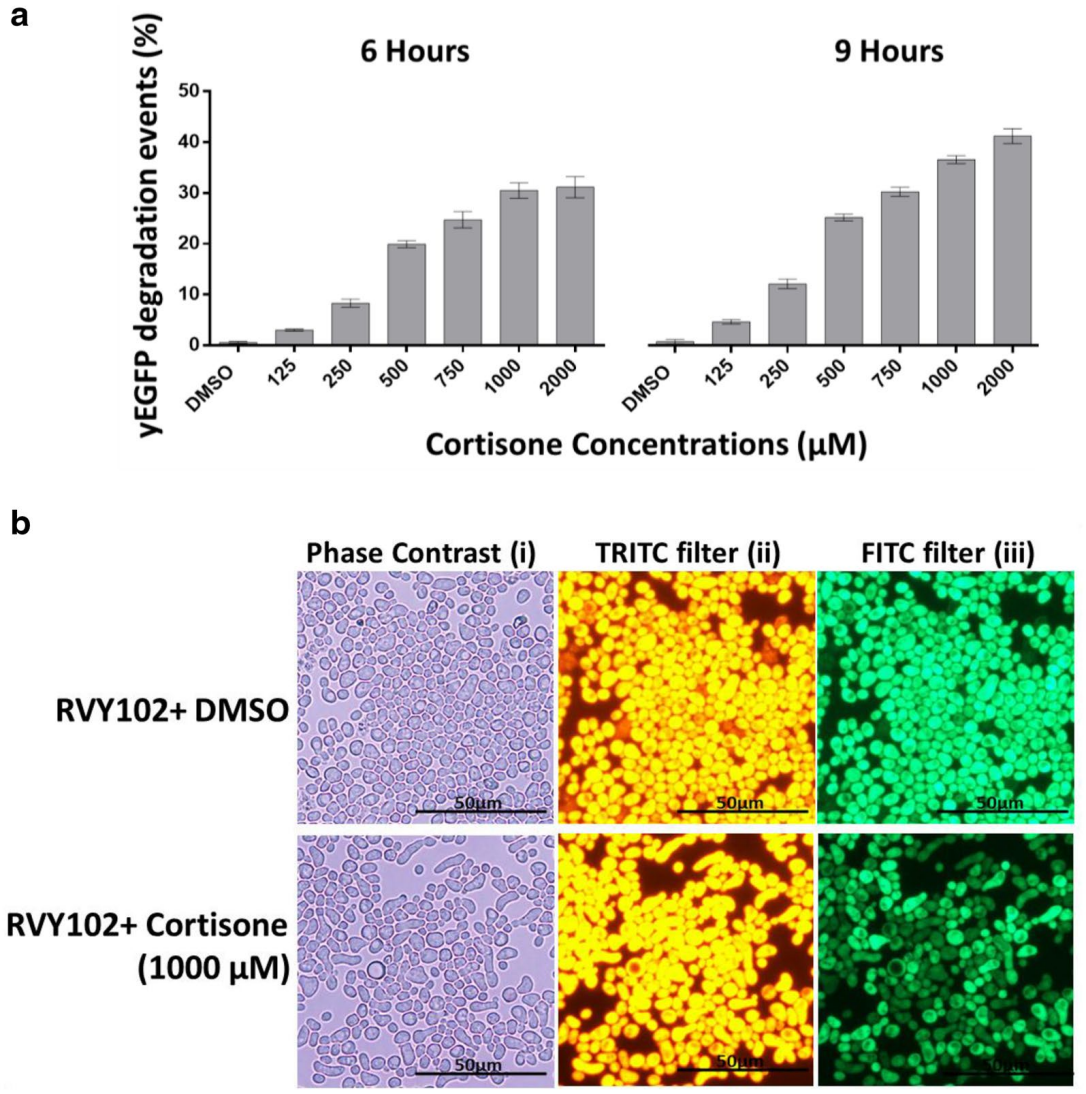
Effect of cortisone on the yEGFP degradation in yeast strain RVY102. **a** Increased cortisone concentrations cause the expression of TEV protease in a time and concentration dependent manner. The TEV protease acts on the reporter protein construct causing degradation of the yEGFP. At both time points tested the yEGFP degradation signal in the cortisone-treated cells was increasing with increasing concentrations of cortisone. The treated RVY102 population was analyzed by flow cytometry (10,000 events) and the results are expressed in percentage of RVY102 cells showing loss of green fluorescence due to yEGFP degradation. In all the samples analysed the tdTomato fluorescence was constant and not affected by the treatment with cortisone. Data shown represent means ± standard deviations of three independent experiments. **b** RVY102 yeast population treated with DMSO and 1000 μM cortisone. Pictures acquired 6 h upon initiation of the treatment with cortisone showed degradation of yEGFP in part of the population (*iii*), while the tdTomato signal is constant in both treated and untreated cells (*ii*)

Functionality of assay strain RVY102 was tested by flow cytometric analyses. We monitored the green fluorescence response at different time points upon addition of different cortisone concentrations (from 125 to 2000 μM) (Fig. 5a). As expected, treatments with increasing concentrations of cortisone caused a gradual loss of yEGFP after 6 and 9 h of incubation indicating the reduction of cortisone to cortisol driven by 11β-HSD1 and the consequent expression of TEV protease. Conversely, cells treated with DMSO maintained stable expression levels of the fluorescent fusion protein. Significant yEGFP degradation was already observed upon 6 h of treatment with 1000 and 2000 μM of cortisone, and degradation further increased upon after 9 h of incubation (Fig. 5a). Fluorescence microscopy images also showed the decrease in yEGFP signal, denoting a loss of green fluorescence upon cortisone treatment whereas td-Tomato fluorescence remained constant (Fig. 5b).

To select for the best experimental conditions many factors including cortisone solubility, time of incubation with the hormone and intensity of yEGFP fluorescence in response to treatments were experimentally evaluated. Finally, 6 h of incubation with 1000 μM cortisone were employed for the validation of strain RVY102.

**Fig. 6:**
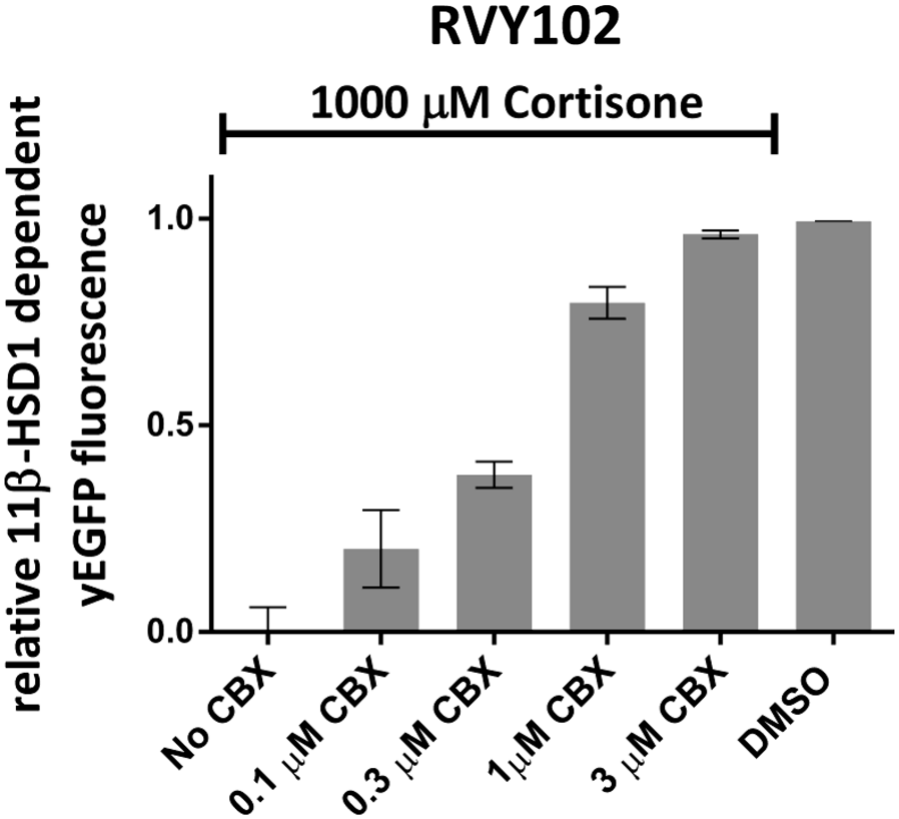
Effect of carbenoxolone (CBX) on 11β-HSD1 dependent response in the strains RVY102. 11β-HSD1 dependent yEGFP fluorescence of strain RVY102 treated with 1000 μM cortisone and increasing CBX concentrations. Increasing concentration of carbenoxolone improved the stability of yEGFP in the yeast population indicating a progressive loss of enzyme activity. Cells treated with 1000 μM cortisone and 3 μM carbenoxolone showed a fluorescence comparable to the one of the untreated population (DMSO) indicating a complete absence of yEGFP degradation. tdTomato fluorescence was maximum and constant in all samples analysed and was not affected by treatments with cortisone and carbenoxolone (not shown). For data normalization details see “Methods” section. Data shown represent means ± standard deviations of three independent experiments

In order to validate the yeast strain RVY102 for screening of putative 11β-HSD1 inhibitors, carbenoxolone concentrations ranging from 0.1 to 3 μM were added to 1 million RVY102 cells together with 1000 μM cortisone. Flow cytometric analyses revealed that increasing concentrations of carbenoxolone gradually led to an increase of the reporter protein in the population. At a concentration of 3 μM carbenoxolone almost no degradation of yEGFP was detected, suggesting complete inhibition of the enzyme 11β-HSD1 (Fig. 6).

### Improving the sensitivity of the assay systems by inhibiting Pdr5p

The relatively high concentration of cortisone required to trigger our yeast based assays, when compared to the concentrations of carbenoxolone needed to completely inhibit 11β-HSD1, raised the question whether the influx of cortisone might be limited, and therefore the intracellular cortisone concentration is much lower than expected. Consequently, we investigated the intracellular availability of cortisone.

**Fig. 7:**
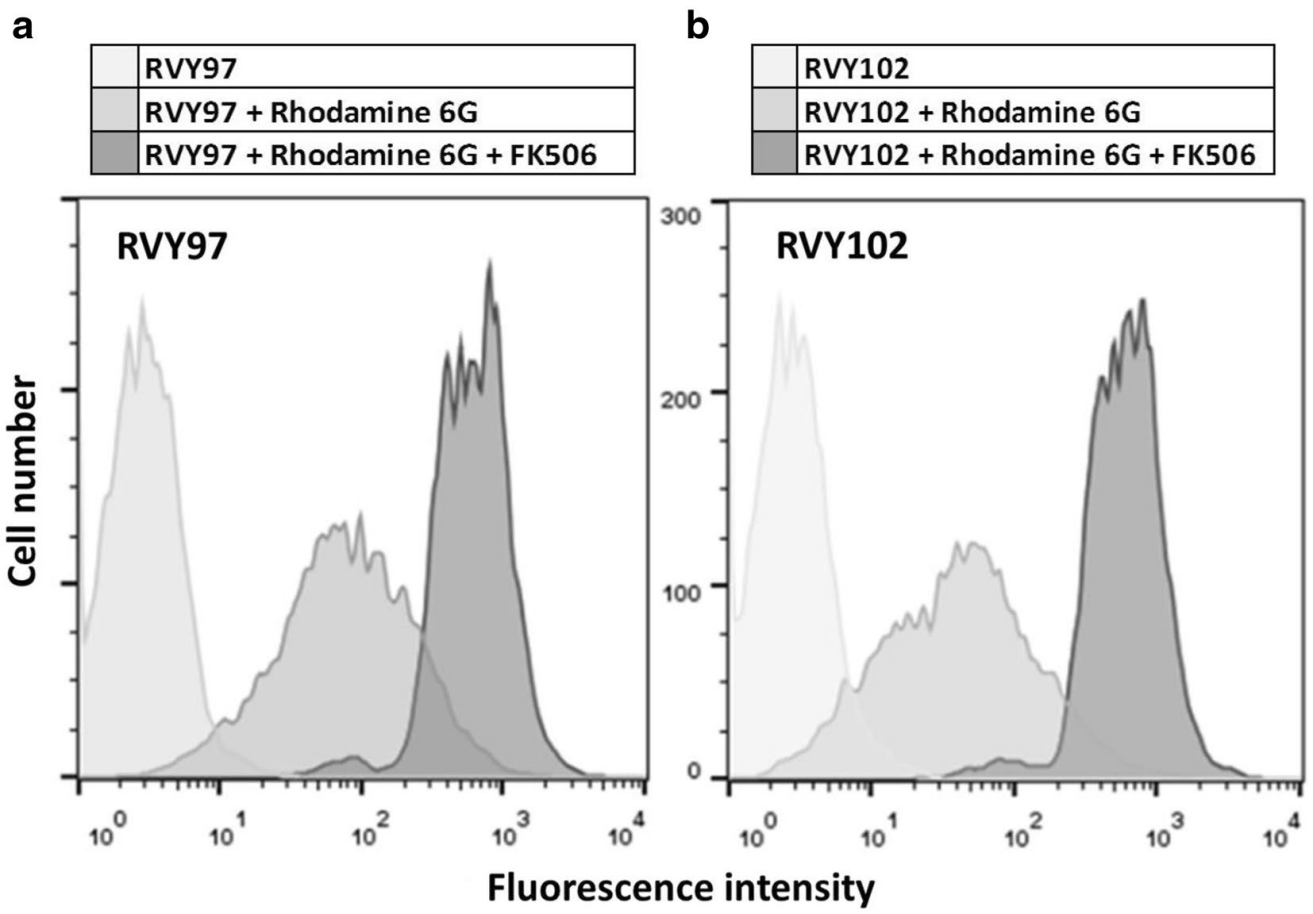
Rhodamine 6G accumulates in yeast cells upon Pdr5p inhibition. Rhodamine 6G accumulation in RVY97 (**a**) and RVY102 (**b**) yeast strains treated or not treated with 10 μg/ml FK506, a known inhibitor of Pdr5p. Each experiment was performed at least three independent times with similar results. The *figure* represents a single result of the replicates

Previous studies demonstrated that in yeast, a low accumulation of certain steroid molecules due to the action of membrane-embedded drug-efflux pumps, can strongly affect the functionality of intracellular cascades driven by an heterologous GR-hormone complex [25, 31]. In the yeast *S. cerevisiae,* an ATP-binding cassette (ABC) transporter, Pdr5p, is able to decrease accumulation of certain steroids, thus changing cellular responses caused by GR-ligand complexes [25]. Pdr5p has many different steroid and non-steroid substrates and tends to protect the cells from an improper accumulation of undesired molecules [32–34]. The null mutants of this transporter allow accumulation of glucocorticoids in cells and maximal activation of the heterologous GR. There is evidence that dexamethasone, a synthetic equivalent of cortisol, is a substrate of the transporter Pdr5p [25]; therefore a possible recognition and excretion of cortisone by Pdr5p was investigated. The expression and activity of Pdr5p in strains RVY97 and RVY102, respectively, was studied using treatment with 5 μM Rhodamine 6G, a well-known target of Pdr5p [34]. Through accumulation of this specific dye within yeast cells, it is possible to get indications about the activity of this transporter. To confirm that accumulation of Rhodamine 6G is limited due to Pdr5p activity, the Pdr5p inhibitor FK506 [35] was added to the cells leading to a strong intracellular accumulation of the dye (Fig. 7). Those experiments demonstrated the presence of significant amounts of active transporter molecules Pdr5p in our assay strains.

**Fig. 8:**
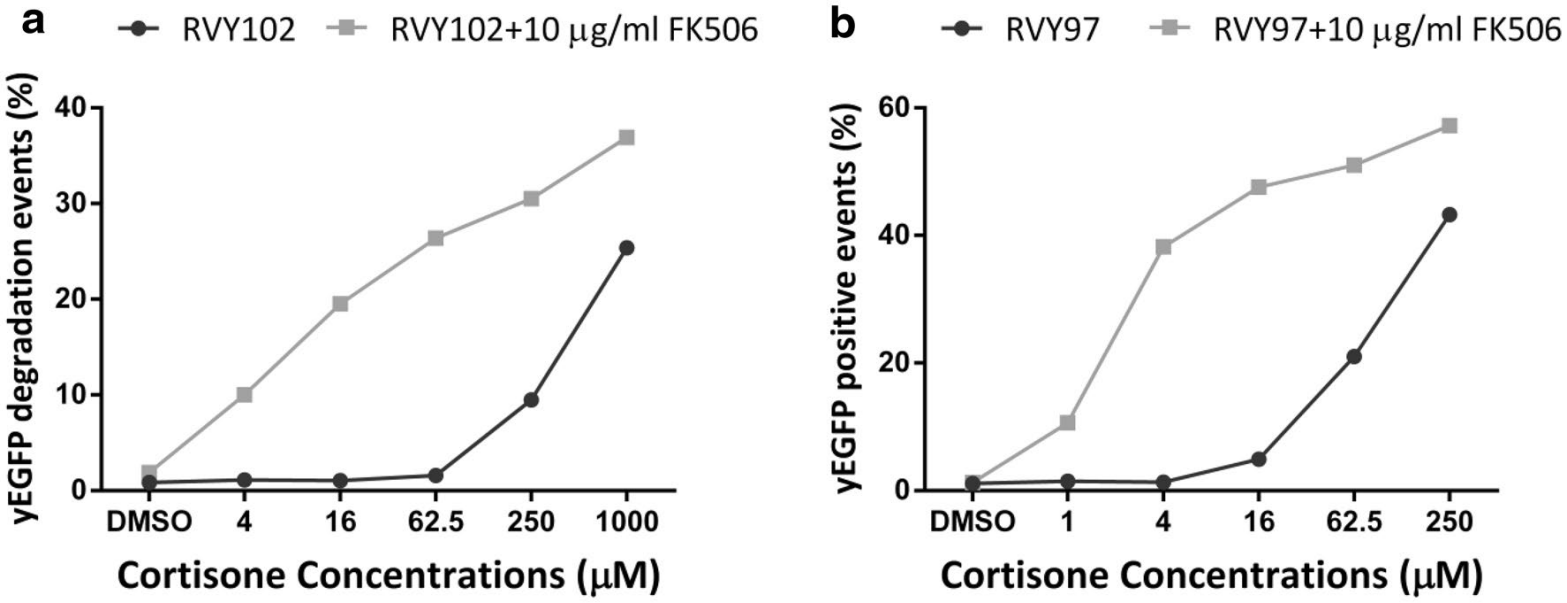
Effects of FK506 on RVY97 and RVY102 assays functionality. Both RVY97 (**a**) and RVY102 (**b**) assays have been tested with or without FK506 at different concentrations of cortisone. In both assays an increased sensitivity to cortisone in presence of the Pdr5p inhibitor is shown. Each experiment was performed at least three independent times with similar results. The *figure* represents a single result of the replicates

Next, we examined the green fluorescent output of strains RVY97 and RVY102 in the presence of 10 μg/ml FK506 and upon treatment with different concentrations of cortisone. Addition of FK506 dramatically improved the sensitivity of the assay strains to cortisone. In fact, hormone concentrations up to 60 times lower than used before caused equivalent responses of the RVY97 and RVY102 strains, when the Pdr5p transporter was not active (Fig. 8). These results strongly suggest that cortisone is a substrate of the Pdr5p transporter.

Membrane transporters, such as Pdr5p in yeast, can impact on whole cell screening assays by, as shown here, limiting the accumulation of effector molecules inside the cells. Therefore, monitoring activity of these transporter molecules can be of crucial importance for the development of efficient whole cell screening assays—essential tools in the early stages of drug development.

For a final validation of the assays, FK506-treated strains RVY97 and RVY102 were incubated with tanshinone IIA, a natural steroid-like compound originally extracted from the roots of *Salvia miltiorrhiza* and recently identified as inhibitor of the human enzyme 11β-HSD1 [36].

**Fig. 9:**
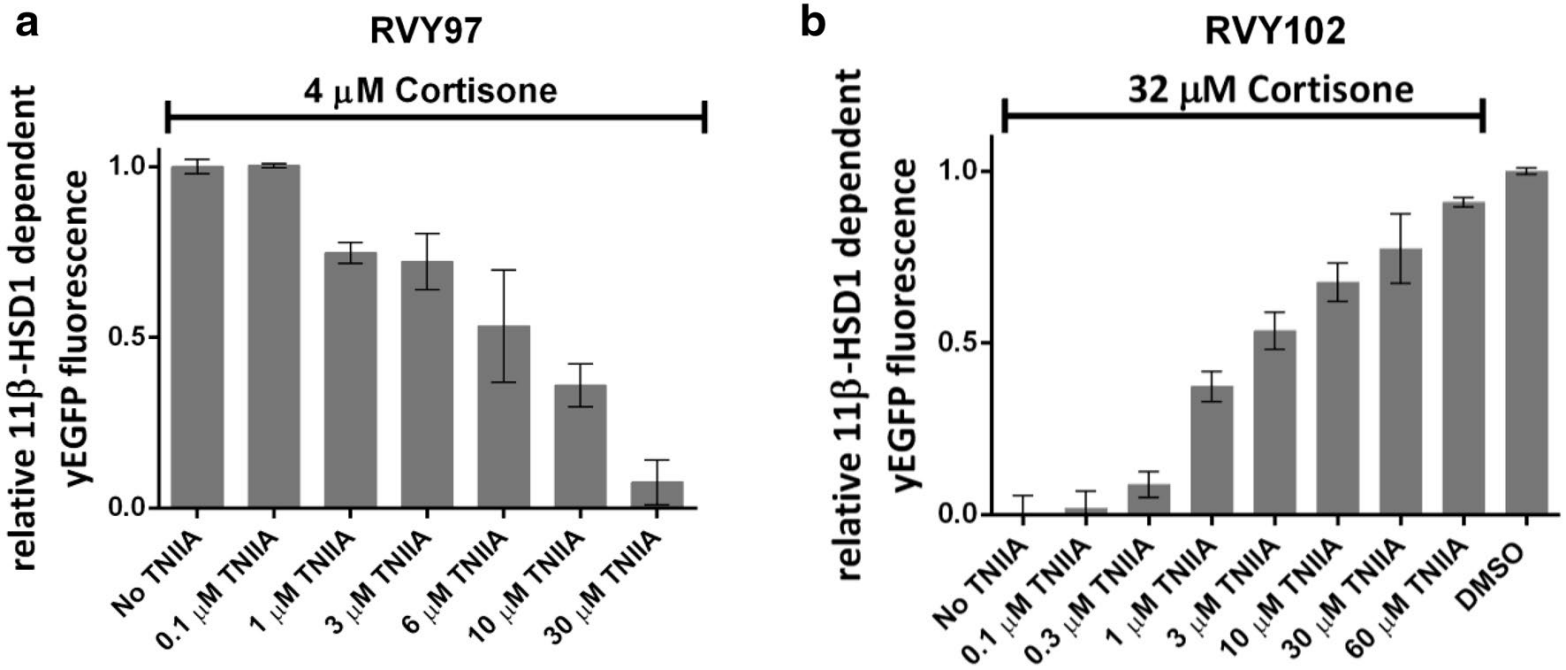
Effect of tanshinone IIA (TNIIA) on 11β-HSD1 dependent response in the strains RVY97 and RVY102, treated with FK506. **a** 11β-HSD1 dependent yEGFP fluorescence of strain RVY97 treated with 4 μM cortisone and with increasing TNIIA concentrations. Increasing concentrations of TNIIA led to a drop of yEGFP *fluorescence* in the yeast population indicating a progressive loss of 11β-HSD1 activity. The fluorescence of untreated cells (no TNIIA) was set at 1. **b** 11β-HSD1 dependent yEGFP fluorescence of strain RVY102 treated with 32 μM cortisone and increasing TNIIA concentrations. Increasing concentrations of tanshinone improved the stability of yEGFP in the yeast population due to the loss of 11β-HSD1 activity. Cells treated with 32 μM cortisone and 60 μM TNIIA showed a fluorescent signal comparable to the one of the untreated population (DMSO) indicating a nearly complete absence of yEGFP degradation. tdTomato fluorescence was constant in all samples analysed, and it was not affected by treatments with cortisone and TNIIA (not shown). For data normalization details see “Methods” section. Data shown in **a** and **b** represent means ± standard deviations of three independent experiments

FK506-treated yeast strain RVY97 was incubated with 4 μM cortisone and increasing concentrations of tanshinone IIA, ranging from 0.1 to 30 μM. After 6 h of incubation, yEGFP fluorescence decreases gradually with increasing concentration of tanshinone IIA indicating progressive inhibition of the activity of human 11β-HSD1 (Fig. 9a).

Similar results were obtained when incubating the FK506-treated strain RVY102 with cortisone and increasing concentrations of tanshinone IIA, ranging from 0.1 to 60 μM (Fig. 9b). The gradual increase of the yEGFP signal with increasing concentrations of tanshinone IIA indicates a progressive inhibition of 11β-HSD1. Those results confirm (a) the inhibitory effect of tanshinone IIA on human 11β-HSD1, and (b) the validity of our yeast-based assays for 11β-HSD1 inhibitor screening campaigns.

## Conclusions

Intracellular glucocorticoid levels play a relevant role in the pathogenesis of metabolic diseases such as obesity and metabolic syndrome. Moreover, high levels of active glucocorticoids in skin have been associated with impaired collagen metabolism and reduced wound healing processes in diabetic and elderly individuals. Therefore inhibitors of 11β-HSD1, the main enzyme responsible for the activation of glucocorticoids at intracellular level, have been proposed as crucial compounds for the treatment of these conditions and for development of skin care products that regulate skin homeostasis. Many strategies to find specific inhibitors for this enzyme have been developed, but only a few molecules have been identified. Among other reasons, this might be due to the fact that the screening of large libraries of small molecules and the identification of pharmaceutically active candidates need very efficient assay systems, often difficult to establish, or whose development is very cost- and labour intense.

The yeast *S. cerevisiae* has become an important eukaryotic host organism for the construction of screening assays using heterologous target enzymes. This work describes the construction of two yeast strains RVY97 and RVY102, functionally expressing the human enzyme 11β-HSD1. The reductase activity of the enzyme on cortisone is efficiently connected with a positive or negative fluorescent signal. The dependency of the output signal on different cortisone concentrations has been demonstrated and its specificity confirmed through testing of the appropriate corresponding control strains, lacking the 11β-HSD1 construct.

During the development of the technology possible causes influencing the sensitivity of the assays to cortisone were considered and the involvement of the yeast drug efflux machinery on the intracellular accumulation of cortisone was demonstrated. The ABC transporter Pdr5p pumps out cortisone, thus preventing its accumulation inside the yeast cells. The inhibition of this transporter, with FK506, significantly increases the sensitivity of the assays.

In order to analyse the suitability of the assay technology for screening candidate 11β-HSD1 inhibitors, the effects of carbenoxolone and tanshinone IIA, two known 11β-HSD1 inhibitors, were investigated in both RVY97 and RVY102 strains. The results clearly show that in both assays the downstream reporter systems responded effectively and with high sensitivity to increasing inhibitor concentrations. A nearly complete inhibition of the human 11β-HSD1 enzyme after treatment with the respective highest concentrations of carbenoxolone and tanshinone IIA was observed.

The yeast-based screening systems described here represent a valid alternative to currently available assays for screening of 11β-HSD1 inhibitors that are mainly based on the use of liver microsomes as source of enzyme, and detection of cortisone and cortisol through liquid chromatography–mass spectrometry [20]. Both yeast assay strains developed, RVY97 and RVY102, are efficient and adaptable primary assay systems for the first steps of a drug discovery process that aims at selecting cell permeable compounds with inhibitory activity for human 11β-HSD1. Due to the nature of the assay certain intrinsic limitations have to be considered. Each compound that displays inhibitory effects somewhere within the assay chain (11β-HSD1, GR, GREs, TEV protease) will pass the first screening gate. In addition, compounds acting on 11β-HSD1 may well be active also on 11β-HSD2, a clearly unwanted off-target effect. To minimize operating expenses due to follow-up of inhibitory molecules of low interest, we propose to run potential hits through one of the classical cell- or microsome-based assays. Time and effort of such tightly focused secondary assays will be easier manageable than entire screening campaigns with such delicate mammalian cell-screening systems. The selected candidates can then be used as lead compounds for the generation of chemically-related molecules with (a) stronger inhibitory effects against the target enzyme, (b) improved metabolic half-life, and (c) low selectivity for other relevant biological targets in order to minimize putative off-target effects.

Moreover, in recent years the use of yeast-based assays in drug discovery processes has been a driving force for the development of innovative hit-finding approaches based on synthetic biology platforms [37]. Advanced combinatorial genetic approaches in baker’s yeast allow for the reconstruction of complex biosynthetic pathways [38]. This enables the yeast cell to synthesise a large variety of chemical scaffolds which, through efficient assay systems, can be directly screened at intracellular level. Here we describe two yeast assay strains, RVY97 and RVY102 that can be easily adapted to be part of a synthetic biology platform in which a diversity of chemical scaffolds is synthetized and immediately screened for activity against the target enzyme 11β-HSD1. Molecules that exert inhibitory activity on 11β-HSD1 will cause a specific intracellular variation of fluorescence thus allowing the identification of those cells that are producing the inhibitor(s). Fluorescence activated cell sorting (FACS) can then be applied to isolate the compound-producing yeast cells.

## Methods

### Chemicals

All chemicals used in this work were purchased from Sigma Aldrich (St. Louis, MO, USA).

### Strain construction and growth conditions

All *S. cerevisiae* strains constructed in this work (Table 1) derive from the S150-2B strain (MATa, *his3*-*Δ1, leu2*-*3 112, trp1*-*289, ura3*-*52*). Yeast precultures were grown in YP medium plus 2 % glucose (YPD) at 30 °C. The integration plasmids carrying the construct *GRE*-*yEGFP* and *GRE*-*TEV* were provided by Evolva SA. The *TEV* and *yEGFP* genes are under the control of a *3xGRE* + *CYC1*-*TATA* promoter that efficiently activates downstream reporter constructs in yeast upon binding the glucocorticoid receptor [39, 40]. In RVY97 and RVY102 strains, the respective *GRE*-*GFP* and *GRE*-*TEV* constructs were integrated as a single copy into the ECM3 locus of chromosome XV of the yeast genome. Positive transformants were selected on YPD agar plates containing Hygromycin (200 μg/ml). In the RVY102 assay strain, the N-degron fluorescent reporter protein construct (Evolva SA), controlled by *GAL1* promoter was integrated as a single copy into the genome at the KIN1 locus of chromosome IV. The positive transformants were selected on YPD agar plates containing Nourseothricin (1 mg/ml). All integration processes were carried out using the lithium acetate transformation protocol [41] and successful integration confirmed by PCR. Two plasmids carrying the murine *Nr3c1* [GenBank: NM_008173] cDNA and the wild type *HSD11B1* [GenBank: NM_005525] human cDNA codifying for GR and 11β-HSD1 respectively were provided by Evolva SA. Both sequences were amplified by PCR and cloned via TA cloning in pYES 2.1 (invitrogen) downstream of the *GAL1* promoter. The *HSD11B1* cDNA was cloned without stop codon and in frame with a sequence coding for a polyhistidine tag. The *GAL1*-*HSD11B1* expression cassette was then amplified by PCR from the pYES 2.1 vector and sub-cloned into a plasmid with a *TRP1* auxotrophic marker (pEVE2113) whereas *Nr3c1* was kept in the pYES 2.1 (*URA3*). The transformation of both ectopic plasmids carrying the *Nr3c1* and *HSD11B1* cDNAs was performed using the lithium acetate transformation protocol. Positive clones were selected on SD agar plates (2 % glucose) lacking uracil and tryptophan for double plasmid selection. All the genetic constructs used for strain construction were confirmed by sequencing.

**Table 1:** Genotypes of the yeast strains assembled in this work

Strain	Genotype
RVY97	S1502B-*ECM3::pCYC 3xGRE*-*yEGFP*-*tADH1 loxP*-*HygR*-*loxP [HSD11B1*-*URA3*-*2μ] [Nr3c1*-*TRP1*-*2μ]*
RVY102	S1502B-*ECM3::pCYC3xGRE*-*TEV*-*tADH1 loxP*-*HygR*-*loxP KIN1::GAL1*-*Degron*-*tADH1 loxP*-*NatR*-*loxP* *[HSD11B1*-*URA3*-*2μ]* *[Nr3c1*-*TRP1*-*2μ]*

All assay experiments with strains RVY97 and RVY102 were carried out in SD medium (2 % glucose) without uracil and tryptophan for 24 h and then washed and re-suspended in SD medium (2 % galactose) without uracil and tryptophan for induction of GR and 11β-HSD1 expression.

### Validation of the assays with carbenoxolone

Yeast strains were maintained for 24 h in 2 % glucose SD medium without uracil and tryptophan. After 24 h, the cells were diluted to an optical density measured at a wavelength of 600 nm (OD_600_) of 0.1 in 2 % galactose SD medium lacking uracil and tryptophan. The cultures were grown for an additional 18 h (about three duplications) in galactose to induce 11β-HSD1, GR and the N-Degron fluorescent fusion protein expression and then divided into 3 ml subcultures. Cortisone was added to the cultures from a 28 mM stock solution in DMSO to the required concentrations. At different time points the cells were collected, resuspended in 1 × PBS and analysed using a FACSCalibur flow cytometer from Becton Dickson (San Jose, CA, USA). Every experiment was repeated independently at least three times.

The specificity of the signal upon treatment with cortisone was confirmed with strains in which the plasmids carrying the 11β-HSD1 expression cassette were replaced by the equivalent empty plasmids. Except for the missing 11β-HSD1 expression construct, the strains were identical to the two parental assay strains RVY97 and RVY102. In the validation of the assays the 11β-HSD1 inhibitor carbenoxolone (CBX) (stock solution 1 mM/water) was added to 10^6^ cells together with cortisone to reach a final concentration of 0.1, 0.3, 1.0 and 3.0 μM, respectively. Cells were incubated for 6 h and after washing in 1 × PBS the samples were analysed by flow cytometry. Both RVY97 and RVY102 strains were tested with carbenoxolone under identical conditions.

Figure 6 shows the green fluorescence of RVY102 samples treated with 1000 μM cortisone and increasing concentration of carbenoxolone (CBX). The percentage of yEGFP degradation events obtained upon treatment of RVY102 cells with 1000 μM cortisone and no carbenoxolone was set at 1. This value was used as reference for the normalization of the other degradation values. In order to get the fluorescence values plotted, the formula “fluorescence = 1 − degradation value” was used.

### Analysis of yeast fluorescence

250 μl of yeast culture volume were centrifuged and re-suspended in 500 μl 1 × PBS. Flow cytometric analyses were performed using the FACSCalibur instrument. The instrument settings were as follows: log forward scatter (FSC) E00; log side scatter 2 (SSC) at 458 V, log FL1 fluorescence at 468 V and log FL2 fluorescence at 460 V. The yEGFP fluorescence was excited at 488 nm and collected through 530/30 nm band-pass filter on the FL1 channel. The tdTomato fluorescence was excited at 488 nm and collected through 585/42 nm band-pass filter on the FL2 channel. For multicolour analysis of the strain RVY102, fluorescence compensation (FL2 20.8 % FL1) was manually set using single colour cell controls. The typical sampling rate was 200 events/s, and the typical sample size was 10,000 cells per measurement. The data were analysed with FlowJo vX.0.7 software. For fluorescence images the samples were taken at different dilutions and examined for green and red fluorescence under the optical microscope BX41 (Olympus) equipped with FITC and TRITC filter sets using a 40x magnification lens and an exposure time of 1/3 s.

### Rhodamine 6G accumulation and efflux

The strains RVY97 and RVY102 (OD_600_ of 0.1) were incubated in the presence of FK506 (10 μM) or DMSO (control) for 20 min followed by incubation with 5 μM Rhodamine 6G for 1 h at 30 °C. 10,000 cells of each population were analysed by flow cytometry.

### Sensitivity to cortisone in presence of FK506

The 11β-HSD1 assay strains RVY97 and RVY102 were tested for the cortisone induced gene transactivation after incubation with 10 μM FK506 or DMSO for 20 min at 30 °C.

### Validation of the assays with tanshinone IIA

RVY97 and RVY102 strains were maintained for 24 h in 2 % glucose SD medium without uracil and tryptophan. After 24 h, the cells were diluted to an OD_600_ of 0.1 in 2 % galactose SD medium lacking uracil and tryptophan. The cultures were grown for an additional 18 h (about three duplications) in galactose to induce 11β-HSD1, GR and the N-degron fluorescent fusion protein expression and then divided into 3 ml subcultures containing 10^6^ cells each. After treatment with FK506 (10 μM) for 20 min at 30 °C, the 11β-HSD1 inhibitor tanshinone IIA (TNIIA) (stock solution 500 μM/methanol) was added to RVY97 cells together with cortisone (4 μM) to reach a final concentration of 0.1,1, 3, 6, 10, 30 μM respectively. The yeast strain RVY102 after incubation with FK506 (10 μM) for 20 min at 30 °C was treated with 32 μM cortisone and tanshinone IIA (TNIIA) (stock solution 500 μM/methanol) at final concentration of 0.1, 0.3, 1, 3, 10, 30, 60 μM respectively.

Cells were incubated for 6 h and after washing in 1 × PBS the samples were analysed by flow cytometry.

The normalization of the data shown in Fig. 9a, b was performed as described in “Validation of the assays with carbenoxolone”.

## Abbreviations

11β-HSDs: 11β-hydroxysteroid dehydrogenase enzymes
11β-HSD1: 11β-hydroxysteroid dehydrogenase 1
11β-HSD2: 11β-hydroxysteroid dehydrogenase 2
GC: glucocorticoid
GR: glucocorticoid receptor
GRE: glucocorticoid response element
yEGFP: yeast enhanced green fluorescent protein
tdTomato: tandem dimer tomato
TEV: tobacco etch virus
TNIIA: tanshinone IIA
DMSO: dimethyl sulfoxide
CBX: carbenoxolone
